# Spontaneously induced prophages are abundant in a naturally evolved bacterial starter culture and deliver competitive advantage to the host

**DOI:** 10.1186/s12866-018-1229-1

**Published:** 2018-09-24

**Authors:** Svetlana Alexeeva, Jesús Adrián Guerra Martínez, Maciej Spus, Eddy J. Smid

**Affiliations:** Laboratory of Food Microbiology, Wageningen University, Wageningen, The Netherlands

**Keywords:** *Lactococcus lactis*, Prophage, Bacteriophage, Lysogeny, Mixed culture, Evolution

## Abstract

**Background:**

In complex microbial ecosystems such as the marine environment, the gastrointestinal tract, but also in mixed culture fermentations, bacteriophages are frequently found to be a part of the microbial community. Moreover, prophages or prophage-like elements are frequently identified in sequenced bacterial genomes. The mixed undefined starter cultures represent an ecosystem which is shaped by long term evolution under relatively defined environmental conditions and provides an interesting model to study co-evolution of phages and their hosts as well as the impact of diversity on microbial community stability.

**Results:**

In the present study we investigated the presence, identity and behaviour of prophages in lactococci being part of a complex cheese starter culture. Genome analysis of representative strains of the 7 genetic lineages of *Lactococcus lactis* constituting the culture indicated the presence of prophages in all strains. Exposure of potential lysogens to mitomycin C confirmed the release of ~ 10^10^·ml^− 1^ phage particles from all tested strains. Furthermore, phages were also released in substantial amounts due to spontaneous induction: more than 10^8^·ml^− 1^ phage particles were present in cultures under non-inducing conditions. This observation suggests continuous release of phage particles by the lactococci. The released bacteriophages exhibited an unusual morphology. For most strains tested, tailless icosahedral phage heads were found. The competitive advantage of lysogens compared to their cured derivatives and their high abundance in the culture suggests that the released tailless bacteriophages play an important role in the ecosystem.

**Conclusions:**

The results of this study indicate that chromosomal genetic elements are active participants in the stable complex microbial community of the starter culture. We show that prophages are abundant in such a community, are produced continuously in large amounts and, despite the huge metabolic burden imposed on the cells by phage particle production, provide a selective advantage to the host.

**Electronic supplementary material:**

The online version of this article (10.1186/s12866-018-1229-1) contains supplementary material, which is available to authorized users.

## Background

Artisanal starter cultures for the production of cheese have a long history of use in a dairy environment and can be considered as domesticated cultures [1]. For that reason, these cultures represent an interesting model ecosystem shaped by relatively defined environmental conditions. Such dairy cultures consist of undefined mixtures of lactic acid bacteria (LAB), usually with *Lactococcus lactis* as most abundant community member. Recently, Erkus and co-workers [2] described the presence of lytic bacteriophages as active members of the starter culture. Already in late 70s of the twentieth century it was suggested that most dairy starter strains are lysogens, meaning that these bacteria contain bacteriophage genomes integrated into their chromosome, a stage in the life cycle of the bacteriophage which is referred to as prophage [3, 4]. Whole genome sequencing of these domesticated lactic acid bacteria uncovered high incidence of lysogeny [5–7]. This shows that prophages are rather common residents of lactic acid bacterial genomes. The prophages of *L. lactis* belong to the temperate P335 group of the *Siphoviridae* family, a taxon of the order of *Caudovirales* (tailed bacteriophages). They resemble lambdoid phages, are heterogeneous in nature and have genomes with a highly mosaic structure with functional modules exchangeable through homologous recombination [8–10]. Frequently, prophages identified from genomic sequences are considered defective or in a state of mutational decay [11]. Nevertheless the presence of prophages in dairy strains is commonly regarded as a threat because bacteriophages can cause significant bacterial mortality leading to production delays or even product loss [12]. Therefore, lysogenic strains rarely find their way to industrial processes [12], thus the beneficial side of lysogeny seems to be underestimated so far.

Whereas lytic bacteriophages can be regarded as predators of prokaryotes, prophages are considered to have either a parasitic or mutualistic interaction with the host [13]. Prophages may carry beneficial properties encoded in their genomes providing the lysogens a selective advantage over their non-lysogenic counterparts [7, 14–16]. A positive impact of prophages on population fitness has recently been addressed by Bondy-Denomy and Davidson [17]. Such benefits are, for example, super-infection immunity, superinfection-exclusion, or adaptive genes of immediate usefulness, acquired from previous hosts. Prophages can potentially spread such properties within a population, shaping a microbial community [18, 19]. In this respect, complex dairy starter cultures provide an interesting model to study co-evolution of phages and their hosts in the dairy environment.

Recently, the originally undefined mixed starter culture Ur has been characterized in detail [2]. Sequencing of representative strains originating from the Ur culture revealed a considerable part of the genomes is made up of prophage-like elements. In this study we addressed the question if these phage-related genetic elements are active participants in the stable complex microbial community, for instance by triggering fully functional prophages into a replicative, lytic life cycle.

## Methods

### Bacterial strains

A high-resolution amplified fragment length polymorphism (AFLP) methodology was used to achieve the delineation of closely related *Lactococcus lactis* strains originating from previously a undefined mixed starter culture referred to as Ur [2, 20]. Representative strains of *Lactococcus lactis* TIFN1-TIFN7 were used throughout this study. These strains represent single colony isolates from different genetic lineages. The strains were maintained as 15% glycerol stocks at − 80 °C and routinely grown in M17 broth (OXOID) with 0.5% (wt/vol) glucose or lactose addition (OXOID).

### In silico analysis of presence of prophages in genomic sequences

The draft assemblies of genomic sequences of TIFN1–7 [2] were analysed by the prophage-predicting PHAST [21] Web server. This bioinformatics tool provides information on prophage completeness of the predicted phage-associated regions defined according to how many genes/proteins of a known phage the region contained: intact (≥90%), questionable (90–60%), and incomplete (≤60%).

### Cell growth, prophage induction and purification

Overnight cultures in M17 broth were diluted up to different OD_600_ (0.05, 0.1, 0.2 and 0.3) depending on the assays and allowed to grow for 1 h at 30 °C before mitomycin C (MitC) was added (final concentrations of 0.3, 0.5, 1 and 1.5 μg/ml were tested). For control purposes, the same diluted cultures without MitC were used. Incubation proceeded for 6 or 7 h and the turbidity at 600 nm was monitored at 1 h intervals.

For applied environmental stress experiments the overnight cultures were diluted to OD_600_ = 0.05. After 1 h of growth under standard conditions the cells were centrifuged at 5000 x g, 30 °C for 10 min to spin down the cells and the growth medium (supernatant) was discarded. At this point stress conditions were applied to the culture: the cell pellets were re-suspended in the same volume of corresponding media prepared in advance (LM17 (undiluted), 0.4-fold concentration of LM17, 1% NaCl, 2% NaCl, 0.3 μg/ml MitC) and pre-heated to 30 °C in order to minimize temperature stress. For the temperature stress the medium was pre-heated to 34 °C. Incubation of cultures was continued at corresponding temperatures (30 °C or 34 °C) in water bath.

Phage particles were isolated from the culture supernatants 6–7 h after the addition of MitC or after changing growth conditions essentially as described earlier [22]. Bacterial cells and debris were removed by centrifugation 5000×g for 15 min at 4°C. The supernatant was filtered through 0.22-mm pore-size Minisart® high flow PES Syringe Filters (Sartorius, Cat #16532---GUK). The pH of the cleared sterile supernatants was adjusted to ~ 7 and the samples were either directly used for gel electrophoresis or further purified and concentrated by PEG/NaCl: 0.25 volumes of a solution containing 20% polyethylene glycol 8000 (PEG) and 2.5 M NaCl (the final concentration is 4% PEG, 0.5 M NaCl) was added. The mixture was kept at 4°C overnight and then centrifuged at 11,000×g for 1 h to precipitate phage particles. The phage particles were suspended in SM buffer (100 mM NaCl, 8 mM MgSO_4_, 50 mM Tris-Cl (pH 7.5)) in 1/160th of original culture volume. It has been shown [23] that these bacteriophage PEG based precipitation conditions do not co-precipitate chromosomal or other contaminating DNA or proteins.

### Electron microscopy

For negative staining transmission electron microscopy (TEM), 5 μL of concentrated phage sample was applied to a 400 mesh copper grid supplied with a formvar/carbon film and incubated for 5 min. The grid was then stained with 2% uranyl acetate for 1 min and allowed to dry. Grids were observed in a JEOL JEM1011 transmission electron microscope at 80 KV and photographed with a 2Kx2K Veleta digital camera (SIS Olympus).

For cryo-TEM, 300 mesh copper Quantifoil 2/2 grids were exposed to a glow-discharge in air prior to being used. Four microliters of phage solution was applied to each grid. Blotting and vitrification in liquid ethane were carried out with a Vitrobot Mark IV instrument (FEI Co.). Grids were then transferred to a Gatan CT 3500 cryo-stage and analyzed in a JEOL JEM2100 transmission electron microscope. Images were recorded at 200 KV under low-dose conditions with a 4 K × 4 K Gatan US4000 digital camera.

### Assaying the bacteriophage DNA content in samples by gel electrophoresis

Phage capsids were disrupted by incubation of aliquots of either sterile supernatants or concentrated phage suspensions to liberate the phage DNA by incubating the samples with 0.2 volumes of SDS-EDTA dye mixture (0.8% (*v*/v) SDS, 60 mM EDTA, 0.5% bromophenol blue, 0.5% xylene cyanol FF and 40% (*w*/*v*) sucrose) for 5 min at 65 °C. The samples were directly loaded onto agarose gel.

Several electrophoresis runs were performed overnight at low field strength (0.5 V/cm), on 0.3% agarose with 2% agarose supporting layer, in TAE, 20 cm gel length, 20 h (Additional file 1: Figure S1A). Selected samples (Additional file 1: Figure S1B) were resolved using field-inversion gel electrophoresis (FIGE, PIppin Pulse (Sage Science)), 1% Lonza SeaKem® GOLD agarose in 0.5-fold concentration TBE, length of gel 10 cm, using the following instrument settings: 75 V (5 V/cm); A. Forward Time at start of run, 150 msec.; B. Reverse Time at start of run, 50 msec; C. Increment added to A at each step, 30 msec.; D Increment added to B at each step, 10 msec.; E. Increment added to C at each step, 3 msec; F. Increment added to D at each step, 1 msec.; number of steps per cycle 81, total run duration 20 h.

For quantification purposes standard electrophoresis conditions (0.7% TAE agarose containing GelGreen, gel length 15 cm, 3-4 V/cm, 1–3 h) were applied. A single band between 24 and 48 bp was consistently observed in all samples.

Gels were scanned and analysed by blue transilluminator gel documentation system (Uvitec Alliance 4.7 Imager equipped with Safelight table and Safelight emission filter).

### Estimation of phage DNA content

The DNA bands were visualized under blue illumination (Uvitec Alliance 4.7 Imager equipped with Safelight table and Safelight emission filter) and the images were used to estimate the amount of DNA via densitometry by UVIband software. DNA ladders GeneRuler High Range Ladder or λmix 19 (Thermo Scientific) were used to indicate high range molecular weight DNA species (10 to 48 kbp). DNA quantification was achieved by comparison with band intensities of known amounts of bacteriophage λ DNA (Thermo Scientific).

The concentration of phage particles was derived based on the phage DNA concentration assuming an average phage size of 40 kbp and 650 g/mol molecular weight of 1 base pair.

$$ number\ of\ copies=\frac{\left(\  DNA\  amount\ \left[ ng\right]\bullet 6.022\bullet {10}^{23}\left[\frac{1}{mole}\right]\right)}{\left( DNA\  length\left[ bp\right]\bullet {10}^9\left[\frac{ng}{g}\right]\bullet 650\left[\frac{g}{mole\bullet bp}\right]\right)} $$

In PEG/NaCl precipitated samples phage particle concentration in original supernatants was derived after correction for concentration factor.

### Prophage curing

Cultures of *L. lactis* TIFN1, TIFN2 and TIFN4 were exposed to 1 μg/ml mitomycin C (MitC) during 6 h similar to the prophage induction procedure. Subsequently the cultures were diluted (10^5^, 10^6^, 10^7^ times) and plated on M17 medium supplemented by 0.5% lactose. Individual colonies were purified and screened by colony PCR using a primer pair targeting P335 group phage sequence [24].

Absence of the amplified fragment indicated potential loss of prophage DNA from bacterial chromosome. The resulting strains were TI1c, TI2c, TI4c – the prophage cured derivatives of TIFN1, 2 and 4 respectively.

### Plasmid construction

The plasmid pSA100 was generated by inserting an annealed pair of synthetic oligonucleotides encoding CP25 constitutive artificial promoter [25] 5′- TCGACCTTTGGCAGTTTATTCTTGACATGTAGTGAGGGGGCTGGTATAATCACATAGTACTGTTA – 3′ and 5′- GATCTAACAGTACTATGTGATTATACCAGCCCCCTCACTACATGTCAAGAATAAACTGCCAAAGG – 3′ into pSEUDO-GFP [26] between the SalI and BglII sites. Next, the EcoRI/BamHI fragment of pSA100 carrying the CP25 promoter, *gfp* (the gene of the superfolder variant of green fluorescent protein, GFP [27]), and the transcription terminator was subcloned into corresponding sites of pIL253 [28] giving rise to pIL-JK2. pIl-SA07 was derived by inserting gene coding for mCherry from pSA047 [29] between the CP25 promoter and the transcription terminator of pIL-JK2 replacing the *gfp*.

### Transformation

Competent lactococcal cells of strain TIFN2 and TI2c were prepared for electrotransformation in osmotically stable media containing 0.5 M sucrose and glycine to weaken cell wall essentially as described by Holo, H. [30]. For *L. lactis* TIFN1 and its derivative TI1c a modification had to be applied because these strains failed to grow when inoculated directly into media supplemented with glycine at concentrations above 0.5%. The modification of the method implied progressive adaptation to higher glycine concentrations by repeatedly inoculating 1 mL of the overnight culture from the previous day into 10 mL of fresh SGGM17 (M17 medium supplemented with 0.5% glucose, 0.5 M sucrose and glycine) with 0.5% glycine increments until reaching the highest concentration tested (2.25%). Stock cultures of these strains in the highest glycine concentration were prepared in 15% (*v*/v) glycerol and referred to as glycine adapted cells (GAC); the culture stocks of GAC were stored at − 80 °C until further use.

The GAC TIFN1 and TI1c cultures pre-grown overnight in SGLM17 media (M17, 0.5 M sucrose, 0.5% lactose and 2.25% glycine) were re-inoculated into fresh SGL17M and grown to OD_600_ of 0.9. Cells were harvested by centrifugation at 6000×g, 4 °C for 20 min and washed once in 1 volume ice-cold washing buffer (0.5 M sucrose, 10% glycerol). A second washing step was performed in 0.5 volume ice-cold EDTA washing buffer (0.5 M sucrose, 10% glycerol and 0.05 M EDTA), followed by a third washing step in 0.25 volume ice-cold washing buffer. Then the cells were resuspended in 0.01 volume washing buffer. Aliquots of 40 μl were used directly for transformation or stored at − 80 °C.

Each aliquot of competent cells was mixed with 1–2 μl (500 ng) of plasmid DNA and then transferred to an ice-cooled electroporation cuvette (2-mm electrode gap) and exposed to a single electrical pulse. The pulse was delivered by the Gene Pulser Xcell Electroporation Systems (Bio-Rad) at 2500 V, 25 μF, 200 Ω resulting in time constants of 4.5 to 5 ms. Immediately following the discharge 1 ml cold recovery medium (M17, 0.5 M sucrose, 0.5% lactose, 20 mM MgCl_2_ and 2 mM CaCl_2_) was added. The cuvette was kept on ice for 5 min and then incubated 2 h at 30 °C. The cells were spread on selection plates (LM17, 1.5% agar, 0.5 M sucrose and 3 μg/ml erythromycin) and incubated at 30 °C for two days. The procedure resulted in transformation efficiencies 1.3·10^4^ and 3.3·10^3^ CFU·μg^− 1^ DNA for TIFN1and TI1c respectively. All strains were verified for its identity and the presence/absence of prophage by strain-specific PCR probes (Table 1).

**Table 1:** PCR primers used in this study

target prophage	code	name	Sequence
proΦ1	P1	pro1-Inch5F	GTTGTGGAGACTTGACGCAGCAA
	P2	pro1-Inch5R	CCTTCAAAACCTAGAAAATGGC
	P3	pro1-Inch3F	GGATGAGAAAAACATTCAATTACAAACA
	P4	pro1-Inch3R	CATCAATGAACGTTTATACCCATATTAC
	strain specific	TIFN1&5f	TCGCTGTCATTGGTATCAGC
		TIFN1&5f	CCAAATTCCGCAGTGTTTTC
proΦ2/4	P1	pro2-Inch5F	ATGTTGAAATCAATGACGACTCAGTT
	P2	pro2-Inch5R	CACGTTACTAAAAAGGCAAAAAAAGAT
	P3	pro2-Inch3F	CGTCGATTGAATGCGAATAAACTAT
	P4	pro2-Inch3R	CAATATACTGTCACACACTCAAAGAC
	strain specific	TIFN2&4f	TGGCTTAGTATTGGCACCTCA
		TIFN2&4r	AGCTGTTCGACCGACACTTT

### PCR

Colony PCR was carried out on bacterial cells, grown on LM17 agar plates, by using DreamTaq DNA polymerase (Thermo Scientific) in 25 μl total volume. The PCR cycling program consisted of 95 °C for 3 min, then 25 cycles of 30 s at 95°C, 1 min at 53°C, and 1 min at 72°C, with an additional step of 5 min at 72°C. PCR primers used are listed in Table 1, the strain specific primers, targeting genetic markers specific for TIFN(1&5) and TIFN(2&4) were as described earlier [2].

### Competition experiment

Overnight cultures of transformed strains: TIFN1/pIL-JK2, TIFN1/pIL-SA07, TI1c/pIL-JK2, TI1c/pIL-SA07 were each diluted to OD_600_ 0.01 with fresh LM17 supplemented with 5 μg/ml erythromycin and mixed pairwise 1:1 in four different combinations (in duplicate). Wild type and cured strains were mixed in reciprocal combinations: GFP-labelled wild type with mCherry-labelled cured strain (TIFN1/pIL-JK2 and TI1c/pIL-SA07) and mCherry-labelled wild type with GFP-labelled cured strain (TIFN1/pIL-SA07 and TI1c/pIL-JK2). Control cultures included the mix of GFP- and mCherry-labelled both wild type strains (TIFN1/pIL-JK2 with TIFN1/pIL-SA07) and GFP- and mCherry-labelled cured derivatives (TI1c/pIL-JK2 with TI1c/pIL-SA07). The mixed cultures were daily propagated by diluting 1% of the overnight culture with fresh medium during 5 days. Ratio between GFP and mCherry expressing cells in samples from day 0, 1, 2 and 5 were analysed by flow cytometry. At day 2 the cultures were also spread on LM17 plates supplemented with 5 μg/ml erythromycin and the ratio between green (GFP) and red (mCherry) colonies in the four mixed cultures was quantified in images acquired using fluorescence Imager UVITEC Alliance 7 series (UVITEC, Cambridge, Great Britain), equipped with Blue excitation light source and emission filter 565 nm for detection of the GFP and Red excitation light source and emission filter 695 nm for detecting mCherry.

### Flow cytometry

Samples were analysed by using a BD FACS Aria™ III flow cytometer (BD Biosciences, San Jose, CA). The cytometer was set up using a 85 μm nozzle and was calibrated daily using BD FACSDiva Cytometer Setup and Tracking (CS&T) software and CS&T Beads (BD Biosciences). 488 nm, air-cooled argon-ion laser and the photomultipliers with 488/10 band pass filter for forward and side scatter and with filter 530/30 nm (with 502 LP filter) for the detection GFP were used. mCherry was excited with yellow-green 561 nm laser and detected by a 610/20 nm with LP 600 nm filter. FSC and SSC voltages of 300 and 350, respectively, and a threshold of 1200 on FSC was applied to gate on the bacterial cell population. The stopping gate was set to 10,000 events. Data were acquired by using BD FACSDiva™ software and analysed by using FlowJo flow cytometry analysis software (Tree Star, Ashland, OR).

## Results

### In silico analysis of potentially inducible prophage pool from a dairy mixed starter culture

An in silico analysis of genomic sequence assemblies of the 7 representative *Lactococcus lactis* strains (labelled as TIFN1-TIFN7) was carried out. Each of these strains are representatives of the 7 *L. lactis* genomic lineages found in the starter culture referred to as Ur [2]. Putative prophage DNA sequences were identified using manual genome browsing based on frequent homology of the ORFs in the chromosomal regions to known phage proteins as well as by using a Web-based prophage-predicting tool PHAST (Phage Search Tool, available at http://phast.wishartlab.com/) [21]. Regions identified as “intact” or “questionable” prophage by PHAST algorithm and manual inspection were considered as potential prophages. All strains in our analysis were predicted to be lysogens and carry at least 1 prophage on the chromosome (see Table 2).

**Table 2:** Summary of predicted prophages in 7 sequenced TIFN strains

STRAIN	NAME	REGION LENGTH	COMPLETENESS	MOST SIMILAR	DETAILS
TIFN1	proΦ1_1	56.1Kb	intact	*Lactococcus* phage bIL309	integrase, capsid, tail, portal, terminase, protease
	proΦ1_2	49.2Kb	questionable	*Lactococcus* phage TP901–1	integrase, transposase, terminase, lysin
TIFN2	proΦ2_1	43Kb	intact	*Lactococcus* phage bIL309	integrase, capsid, tail, portal, terminase, protease, lysin
	proΦ2_2	57.7Kb	intact	*Lactococcus* phage bIL309	integrase, tail, portal, terminase, protease
TIFN3	proΦ3	36.8Kb	questionable	–	integrase, transposase
TIFN4	proΦ4_1	44.1Kb	intact	*Lactococcus* phage bIL309	integrase, capsid, tail, portal, terminase, protease
	proΦ4_2	60Kb	intact	*Lactococcus* phage bIL309	integrase, tail, portal, terminase, protease
TIFN5	proΦ5_1	47.5Kb	questionable	*Lactococcus* phage TP901–1	integrase, transposase, terminase
	proΦ5_2	31Kb	intact	*Lactococcus* phage bIL309	integrase, capsid, tail, portal, terminase, protease
TIFN6	proΦ6_1	31Kb	questionable	*Lactococcus* phage TP901–1	tail, transposase, terminase, Lysin
	proΦ6_2	60.7Kb	intact	*Lactococcus* phage bIL309	integrase, capsid, tail, transposase, head, portal, terminase, protease, lysin
TIFN7	proΦ7_2	42.1Kb	questionable	*Lactococcus* phage phiLC3	capsid, transposase, terminase
	proΦ7_3	43.8Kb	questionable	–	transposase, terminase

As anticipated, all predicted bacteriophages were found to belong to the P335 group of *Siphoviridae* phages. Because the chromosomal sequence information consists of separate pseudo-assembled scaffolds, exact prophage locations on the bacterial chromosomes and their correct genetic makeup were difficult to determine.

### Mitomycin C induced phage release

To experimentally confirm the ability of Ur prophages to undergo lysogenic/lytic conversion, exponentially growing cells were exposed to mitomycin C (MitC) treatment. Seven representative strains from the complex dairy culture Ur (strains TIFN1, TIFN2, TIFN3, TIFN4, TIFN5, TIFN6, TIFN7) along with two control *L. lactis* strains IL1403 and *L. lactis* MG1363 were used in this study. *L. lactis* IL1403 can be considered as a positive control since it was shown to possess six prophages related sequences [8]. *L. lactis* MG1363 is taken as a negative control because induction of prophages has never been reported in the literature for this strain [31].

Using supernatants of the induced cultures, attempts have been made to identify sensitive strains within the Ur consortium (54 Ur isolates) as well as among 7 unrelated strains (industrial *L. lactis* isolates, Laboratory of Food Microbiology, in-house culture collection), and *L. lactis* MG1363 and IL1403. All these lactococcal strains were challenged with supernatants derived from the MitC treated cultures in a spot assay. No sensitive host strains could be identified for any of supernatants (not shown) as no plaques were produced on the cell lawns.

**Fig. 1:**
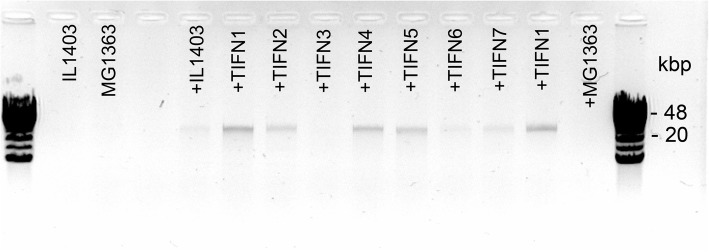
Release of phage size DNA in supernatants of strains treated with Mitomycin C (MitC). Above each lane *Lactococcus lactis* strains TIFN1–7 are indicated along with control strains MG1363 and IL1403, (+) indicate samples subjected to 1 μg/ml MitC treatment (initial OD_600_ was 0.2)

In contrast, phage-size DNA bands (25–45 kbp) were readily visible on agarose gels after treatment with SDS/EDTA in nearly all cleared, filtered supernatants of MitC treated Ur strains (Fig. 1). Upon MitC induction, DNA corresponding to phage DNA size, between 25 and 45 kbp, is readily visible in all samples except in the negative control strain *L. lactis* MG1363 as anticipated, and in *L. lactis* TIFN3. The latter strain showed visible DNA bands only after concentrating the sample (see below), suggesting that this strain released the lowest amount of phage DNA. The size of the bacteriophage genome was analysed more accurately for several samples by either long overnight electrophoresis runs at low field strength or by field-inversion gel electrophoresis (FIGE). Molecular weights of proΦ1 and proΦ5 were estimated at 40.7 kbp and proΦ2 was estimated at 39.5 kbp (Additional file 1: Figure S1). Moreover, 14 additional Ur strains belonging to lineages 1 and 5 [2] were analysed and all of them released detectable amounts of phage DNA upon MitC induction (Additional file 2: Figure S2).

### Phage morphology and dimensions

**Fig. 2:**
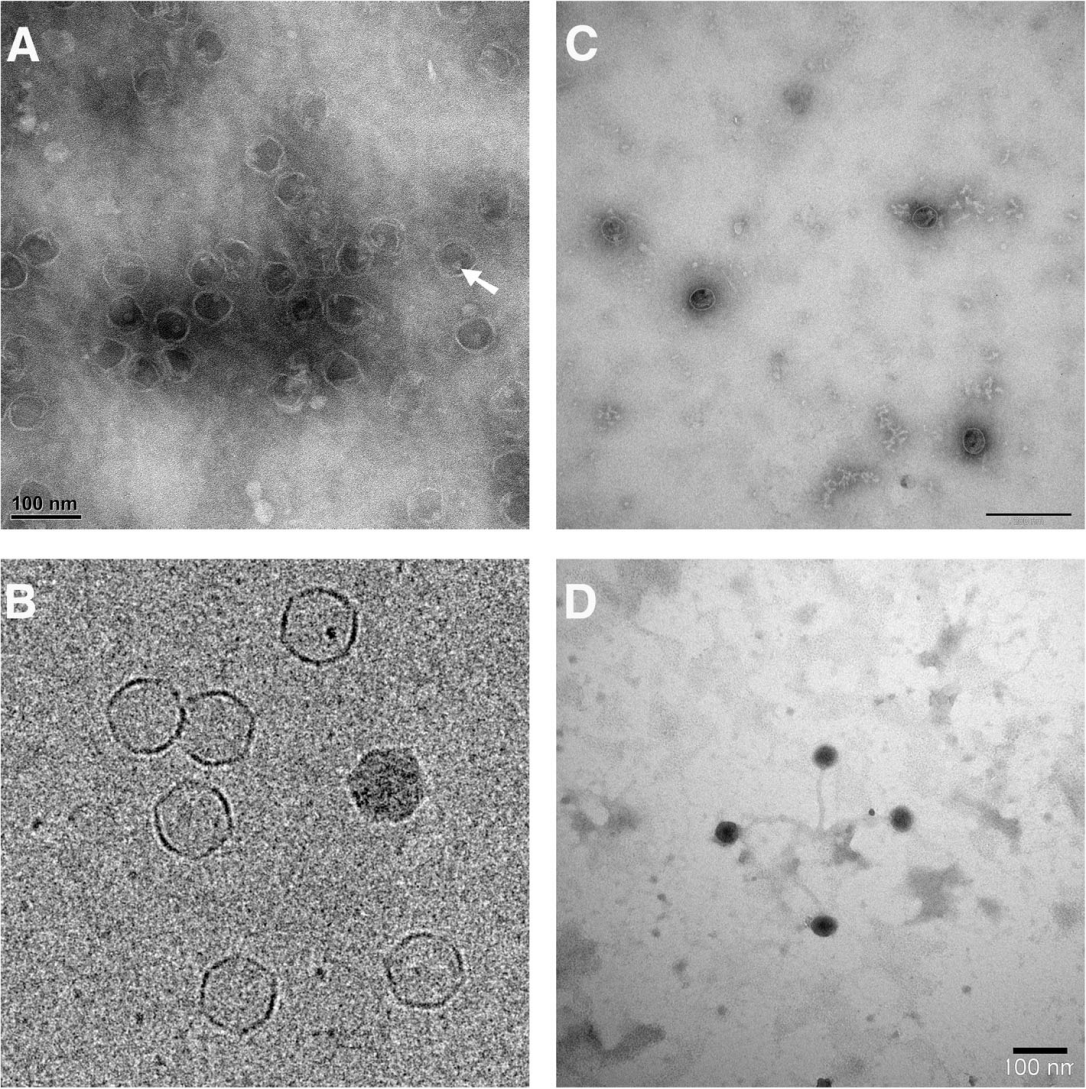
Electron micrograph of the released bacteriophages. **a** Negative (uranyl acetate) staining Transmission Electron Microscopy (TEM) image of a typical bacteriophage (proΦ1). The white arrow indicates a characteristic electron density inside the heads. **b** Cryo-TEM image of proΦ1. **c** Bacteriophage proΦ5, released from TIFN5 under non-inducing conditions. **d** Bacteriophage SK11 with intact tails released from SK11 strain after MitC induction

Electron microscopic examination was performed to confirm that the released large DNA molecules belong to expressed bacteriophages. PEG/NaCl concentrated post-induction supernatants were subjected to microscopic observations after removal of residual PEG by chloroform treatment. A representative electron micrograph of a negatively stained specimen derived from *L. lactis* TIFN1 (designated as proΦ1, see Fig. 2a), shows a capsid of 55 ± 2 nm, which is similar to those of other members of the P335 group phages [32], but lacking a tail structure. We also consistently observed an electron dense spot within the head structure (arrow). This may indicate the presence of a large macromolecular complex in or attached to the capsid. Cryo-electron microscopy (cryo-TEM) performed on the same sample clearly showed icosahedral shaped heads (Fig. 2b). The same tailless heads, albeit in much lower amounts we observed in PEG/NaCl concentrated supernatants from cultures not subjected to MitC induction (uninduced cultures, Fig. 2c). In parallel, positive control culture of the lysogenic *L. lactis* strain SK11 [33] was subjected to MitC induction. Small phages with capsid dimensions 42±2.4 nm and attached long tails ~ 142 nm were observed in the SK11 concentrated supernatants (Fig. 2d). Only one of the analysed strains (*L. lactis* TIFN6) produced, in addition to tailless phage particles, also separated phage tail structures.

### Effect of induction conditions on cell growth and phage release

**Fig. 3:**
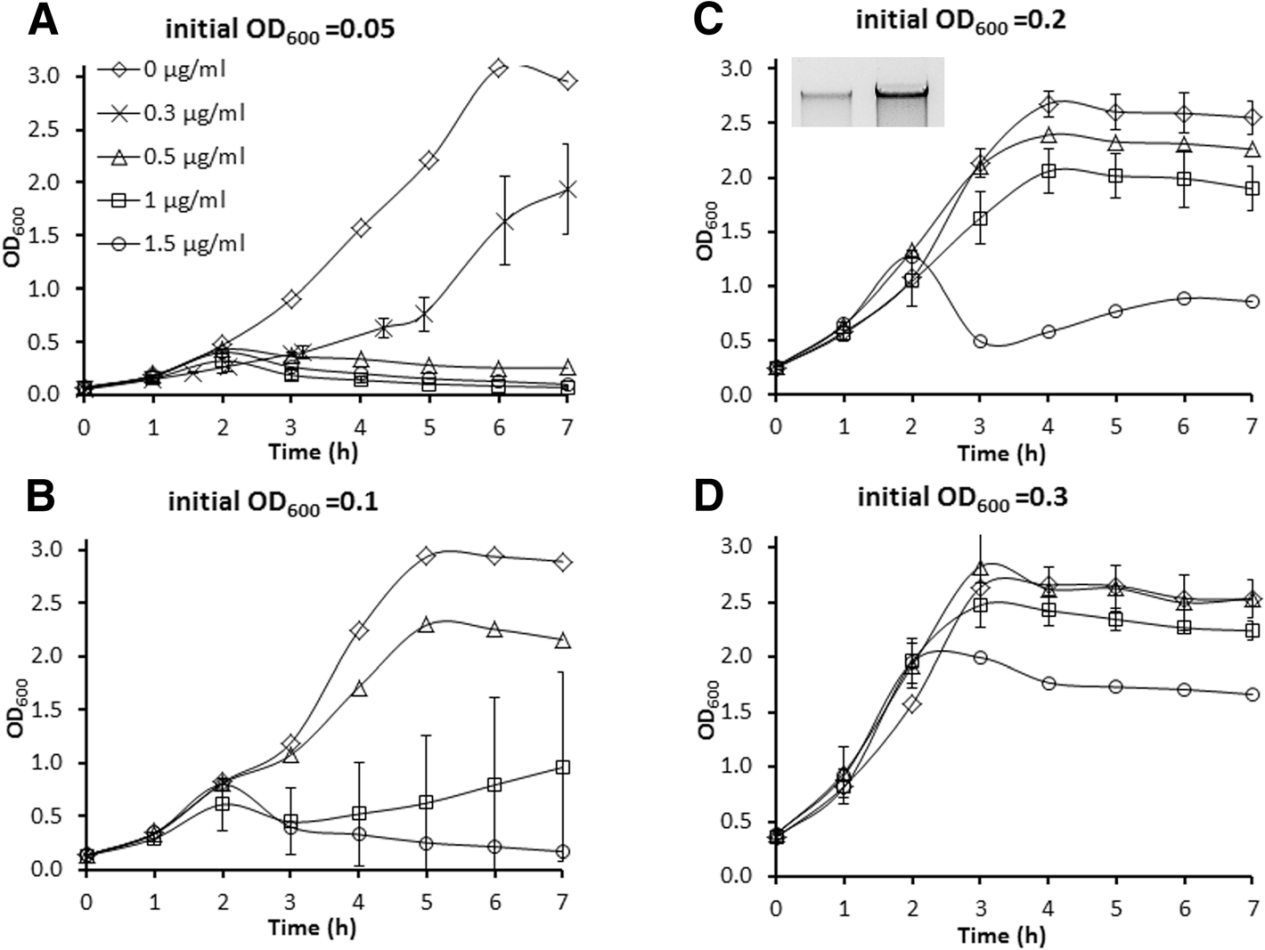
Growth response of *Lactococcus lactis* TIFN1 to different induction conditions. Mitomycin C (MitC) induction of TIFN1 initiated at different growth phase (OD_600_) and by different MitC concentrations. Initial OD_600_ 0.05 (panel **a**), 0.1 (**b**), 0.2 **(c)**, 0.3 (**d**) correspond to OD_600_ ~ 0.1, 0.2, 0.4, 0.6, respectively, at the time point of MitC addition (after 1 h of growth without MitC). In graph C, the insert shows phage DNA release at 7 h of the incubation period (left: spontaneous induction; right: MitC (1 μg/mL)

**Fig. 4:**
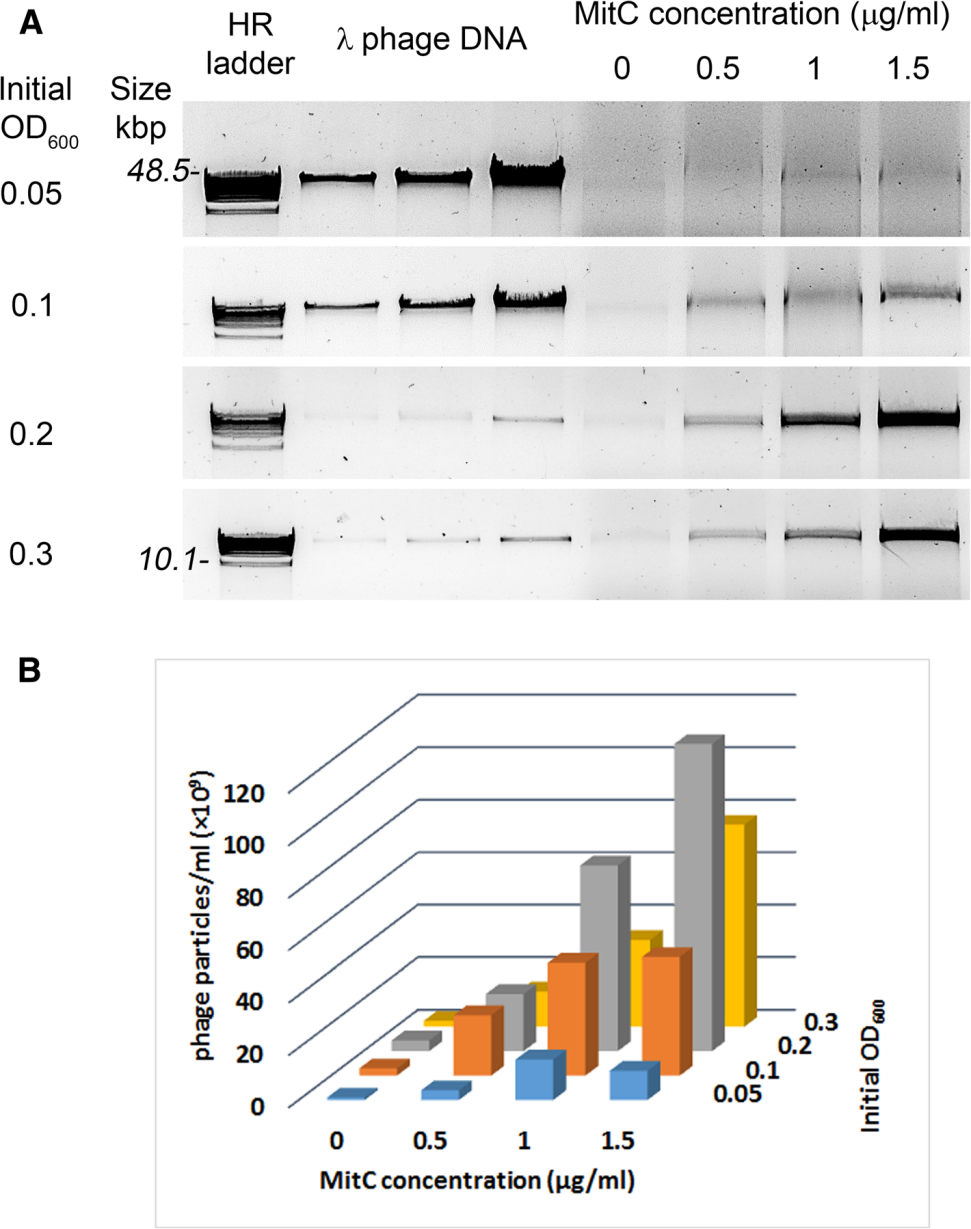
Phage release by *L. lactis* TINF1 at different induction conditions. Initial growth phases (OD_600_) and MitC concentrations were varied. **a** Phage was visualized on agarose gel electrophoresis. HR – high molecular weight DNA marker (10–48 kbp, 250 ng total DNA amount). The amount of λDNA was used as follows: 75, 100, and 175 ng for initial OD_600_ = 0.05 and 0.1; 5, 10, and 25 ng for initial OD_600_ = 0.2 and 0.3. Estimated number of phage particles is shown in panel (**b**)

To shed more light on the process of phage particle production, growth behaviour and phage yield were monitored simultaneously for relevant strains. To evaluate optimal conditions for prophage induction in the Ur strains TIFN1 to TIFN7, we varied the timing of induction during culture growth and studied the release of phage crop as a function of the concentration of the inducer mitomycin C (MitC). Addition of MitC at the onset of exponential phase (initial optical density at 600 nm, OD_600_ = 0.05) strongly inhibited growth of *L. lactis* TIFN1 at all tested MitC concentrations except for the lowest concentration 0.3 μg/ml used (Fig. 3a). We varied the initial culture OD_600_ and added the inducer after approximately 1 doubling (1 h). Increasing initial culture density from 0.05 to 0.3 (or from OD_600_ = 0.1 up to 0.6 at initiation of induction, Fig. 3) resulted in less growth inhibition. This effect inversely correlated with increasing MitC (0.5 to 1.5 μg/ml) concentrations. Analysis of phage crop yield as a function of different conditions (Fig. 4) showed that the induction efficiency is dependent on the growth phase as well as on the inducer concentration. This is in agreement with earlier observations [34]. Initiation of induction at the late exponential phase, OD_600_ = 0.4 or 0.6 (or initial OD_600_ = 0.2–0.3), and elevated MitC concentrations generated significantly higher prophage induction and release of phage crop for all tested lysogenic strains. Some induction conditions resulted in a highly variable cell growth response (e.g. see Fig. 3b). Interestingly, conditions of maximum phage yields neither resulted in culture clearance nor in a decrease in optical density.

**Fig. 5:**
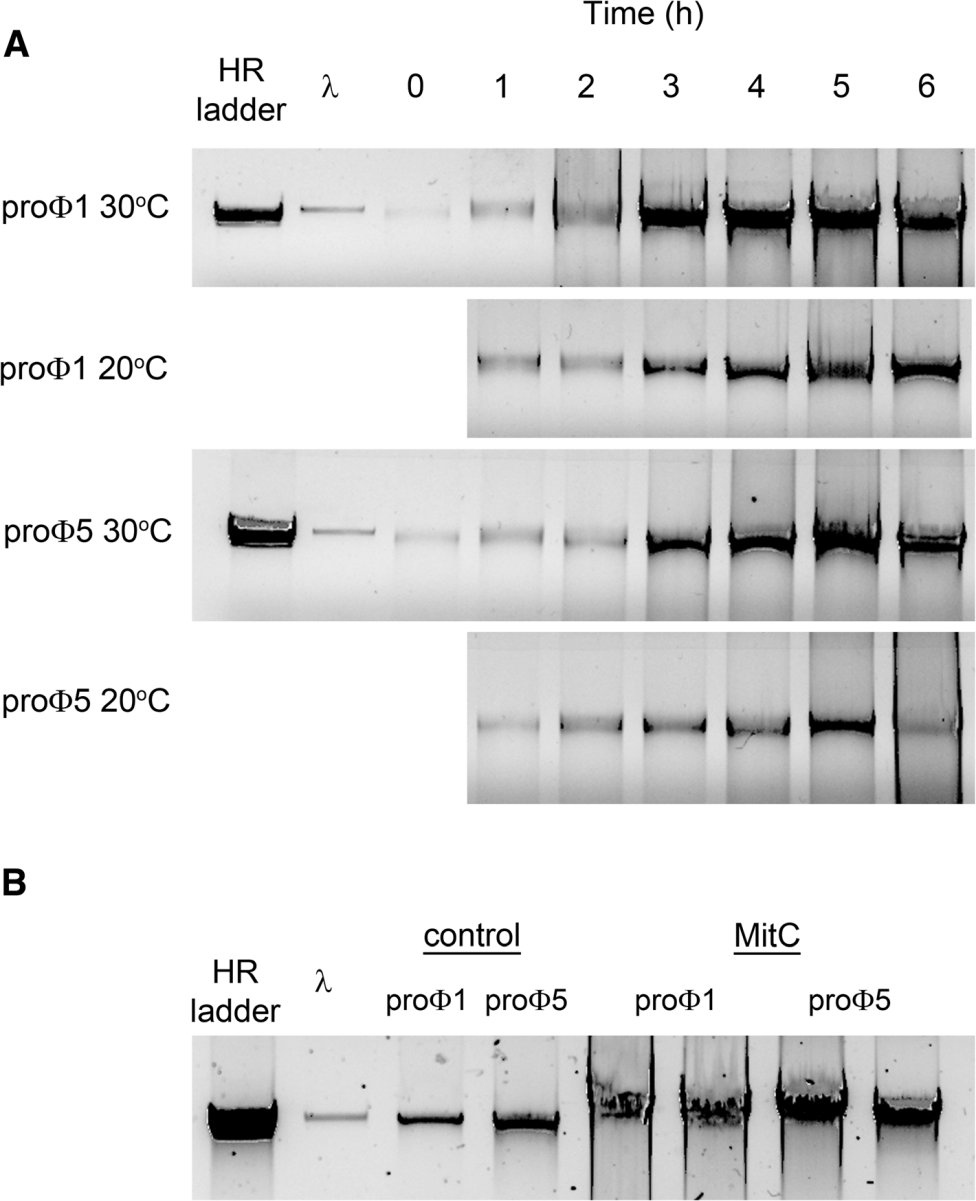
Dynamics of phage release by *L. lactis* TIFN1 (proΦ1) and *L. lactis* TIFN5 (proΦ5). **a** Phage release from cultures (initial OD_600_ = 0.2) after treatment with MitC (1 μg/ml) and incubated at 20 and 30 °C for 6 h was monitored by visualizing the DNA from the viral progeny throughout the incubation period on agarose gel electrophoresis. **b** Phage DNA from non-induced cultures and MitC-induced cultures (in duplicate) of the strains TINF1 and TINF5 at the end of the incubation period (6 h) at 30 °C. λphage DNA (50 ng) is used as reference

Induction dynamics performed for *L. lactis* TIFN1 and *L. lactis* TIFN5 at 20°C and 30°C showed maximum yields of phage crop at 30°C after MitC induction during 5–6 h (Fig. 5a). As expected, induction proceeded at a lower rate at 20°C. Interestingly, the presence of phage DNA was even observed before addition of an inducer (a faint band is shown in Fig. 5a, time 0) in cultures grown at 30 °C. Regarding this, we concentrated phage crops using PEG/NaCl precipitation in order to compare the amount of phage DNA detected in *L. lactis* TIFN1 and TIFN5 cultures after MitC and spontaneous induction at the end of the incubation period (Fig. 5b). Profuse phage release was observed, even in non-induced cultures.

### MitC driven and spontaneous prophage induction in Ur strains

**Fig. 6:**
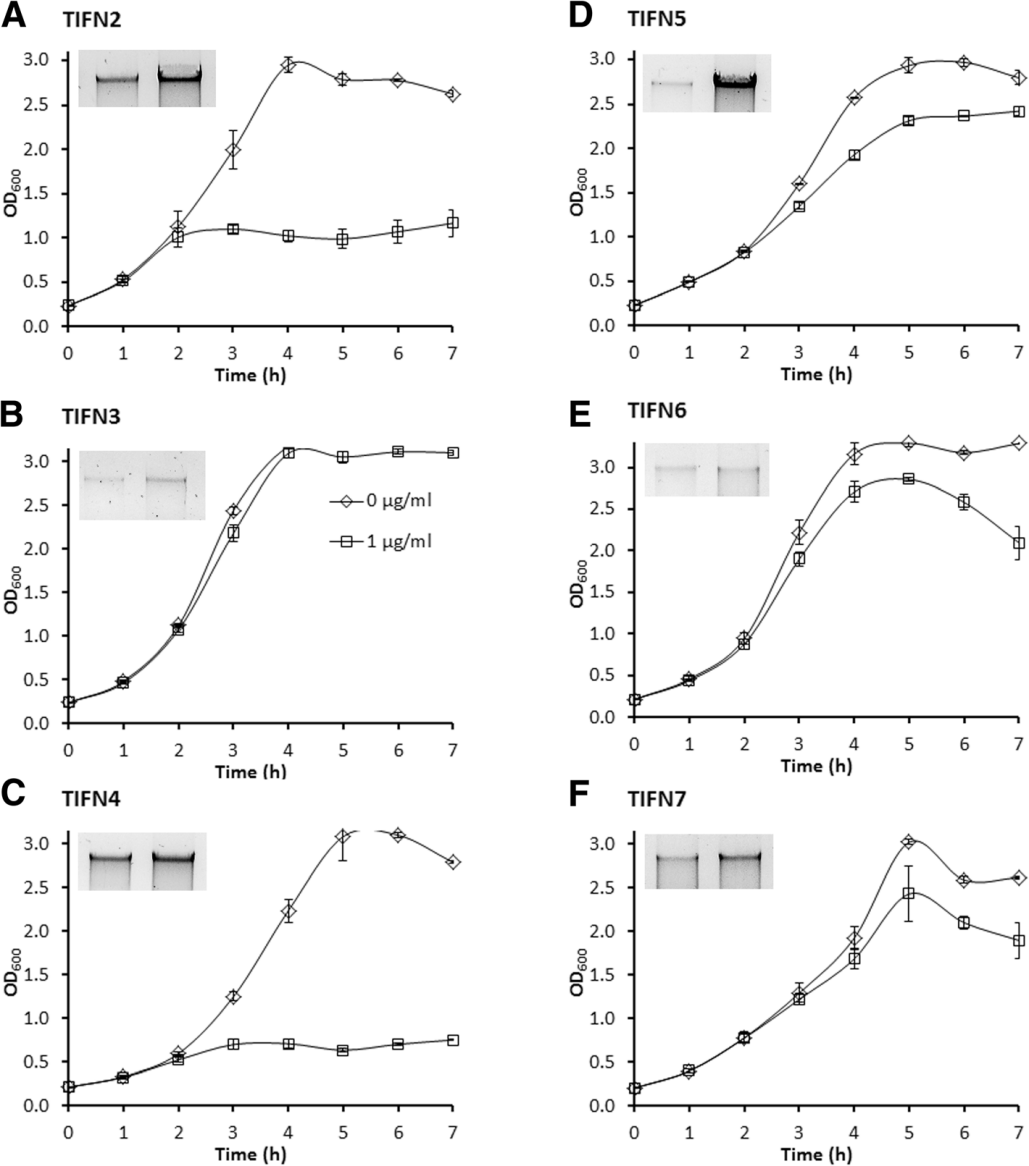
Growth response of L lactis TIFN2 (**a**), TIFN3 (**b**), TIFN4 (**c**), TIFN5 (**d**), TIFN 6 (**e**) and TIFN7 (**f**) to 1 μg/ml MitC induction. The induction was initiated at OD_600_ = 0.4. Initial OD_600_ was set to 0.2 resulting in OD_600_~ 0.4 at the MitC induction start. To control samples (diamonds) no MitC was added. The inserts show phage DNA released at the end of the induction (time point 7 h) for un-induced samples (left) and induced samples (right)

Once the optimal prophage induction conditions were established for strain TIFN1, we proceeded to analyse and compare the growth response and prophage release in the other representative lactococcal strains (*L. lactis* TIFN2, *L. lactis* TIFN3, *L. lactis* TIFN4, *L. lactis* TIFN5, *L. lactis* TIFN6 and *L. lactis* TIFN7) selected from Ur starter culture. MitC was added at a concentration of 1 μg/mL to growing cultures at late exponential phase (initial OD_600_ = 0.2, which corresponded to OD_600_ of ~ 0.4 at the point of MitC addition after 1 h of growth). Induction was continued for 6 h at 30°C. Non-induced cultures (no MitC added) were analysed as controls. The experimental conditions hardly affected growth of most of the strains (Fig. 6). Only cultures of *L. lactis* TIFN2 (Fig. 6a) and *L. lactis* TIFN4 (Fig. 6c), showed a growth arrest upon addition of MitC. Interestingly, the latter two strains belong to the subspecies *lactis* while the others belong to the susbspecies *cremoris*.

**Fig. 7:**
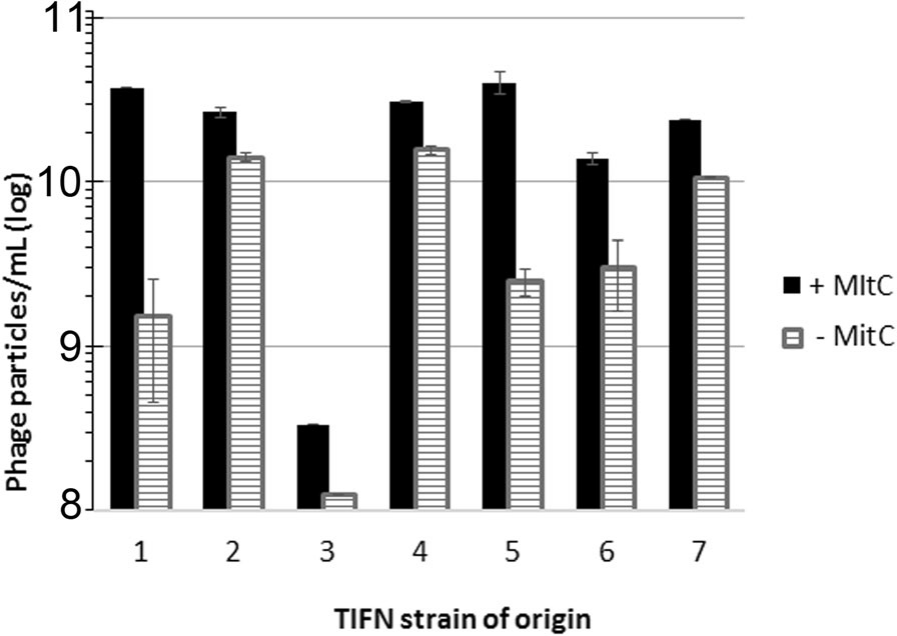
Quantification of phage particles release in the absence and presence of MitC. TIFN1, TIFN2, TIFN3, TIFN4, TIFN5, TIFN6 and TIFN7 *L. lactis* strains were analysed. Control samples (no MitC) indicated by dashed bars; mitomycin C treated samples (+MitC) indicated by filled bars. The quantification is based on quantification of phage DNA by agarose gel electrophoresis

We estimated the phage DNA concentration in the supernatants by densitometric quantification of DNA in GelRed-stained agarose gels using as a reference λDNA (Fig. 7). Determination of the DNA content allowed us to estimate the concentration of phage particles. Spontaneous prophage induction resulted in a phage crop of approximately 10^9^·ml^− 1^ phage particles in 6 out of 7 culture supernatants analysed. One exception is *L. lactis* TIFN3, which produced 10-fold less phage particles in the absence of an inducer. Only after concentrating the sample of this particular strain with PEG/NaCl, we were able to detect a prophage-related DNA band in the gel. Upon addition of MitC the concentration of phages in 6 out of 7 strains increased 2–20 times to approximately 10^10^·ml^− 1^ liberated phage particles.

### Change in spontaneous prophage induction at stress conditions

To determine the potential effect of the environmental stress factors on the spontaneous prophage induction, we challenged exponentially growing cultures (initial OD_600_ = 0.05 with various stress conditions. The mild heat shock (temperature of incubation was suddenly changed from 30 to 34 °C), osmotic up-shock (LM17 broth was supplemented with 1 and 2% *w*/*v* NaCl), and nutrient limitation (cultures were transferred from LM17 broth to 0.4-fold concentration LM17) were applied to cultures at OD_600_~ 0.1. Different responses to the stress conditions were observed in the lysogenic strains (Table 3, Additional file 3: Figure S3 and Additional file 4: Figure S4). Slightly elevated temperature (34°C) resulted in increased spontaneous prophage induction in 5 of the 7 strains tested (*L. lactis* TIFN1, TIFN2, TIFN4, TIFN5 and TIFN7). In *L. lactis* TIFN3 and *L. lactis* TIFN6, increased prophage induction was provoked by increased osmolarity. Interestingly, reduced nutrient availability, in contrast to other conditions resulted in slightly decreased prophage induction.

**Table 3:** Comparative effect of the environmental stress and standard growth conditions on spontaneous prophage induction

phage	MitC	0.4-fold LM17	34 °C	1% NaCl	2% NaCl
proΦ1	+		+		
proΦ2	+	–	+		
proΦ3	+			+	+
proΦ4	+	–	+		
proΦ5	+	–	+		
proΦ6	+	ND		+	+
proΦ7	+		+		

The table summarises data shown in Additional file 3 and Additional file 4. (+) stress induction > standard induction; (−) stress induction < standard induction; (empty cell) not significant differences; (ND) not determined. MitC induction (0.3 μg/ml) was used as positive control

### Prophage curing, lysogen instability and continuous phage excision/DNA replication

**Fig. 8:**
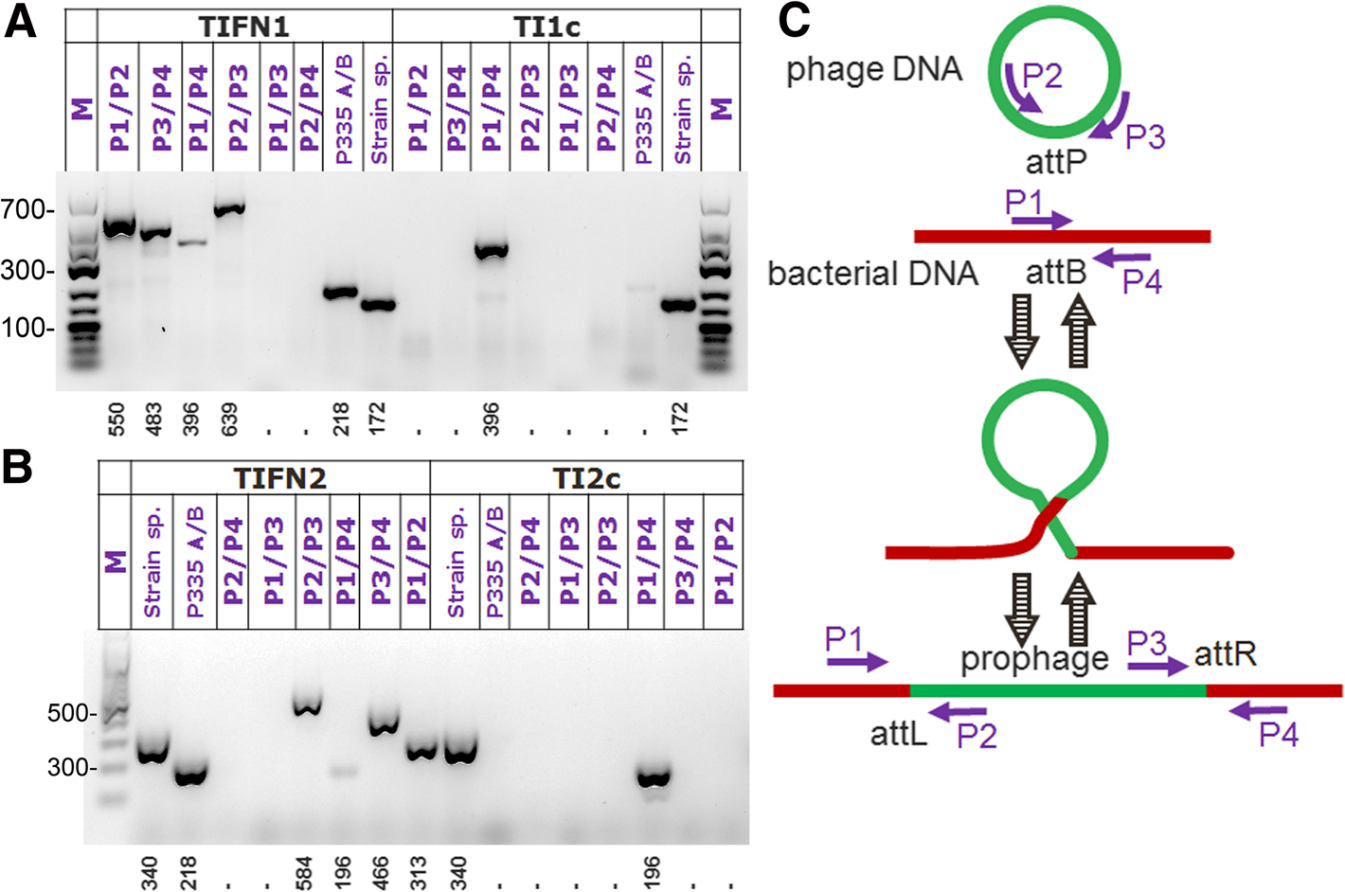
PCR analysis of prophage excision and integration. Panel **a** shows the analysis of *L. lactis* TIFN1 and its cured derivative TI1c. Panel **b** shows the analysis of *L. lactis* TIFN2 and its cured derivative TI2c. The combination of primers are indicated above each lane, expected amplicon size is depicted beneath each lane in panels **a** and (**b**), (**c**). Schematic representation of the PCR analysis strategy showing primer binding sites (not to scale)

To determine the impact of the presence of prophages in the chromosome on the host fitness we isolated prophage-cured derivatives for several strains: TIFN1, TIFN2, and TIFN4. These strains were annotated TI1c, TI2c, and TI4c respectively. During the initial screening we found a high rate of prophage loss (1:30 to 1:60 of the screened colonies were found to be prophage-free), indicating a high degree of prophage excision. The strain identity and the absence of prophages were confirmed by using strain and phage specific PCR primers. Based on the available sequence information we predicted phage ends and their chromosomal insertions and used these predictions to design PCR primers for detecting and discriminating between (i) integrated prophage, encompassing either attR (P1/P2) or attL sites (P3/P4), (ii) circularized prophage (P2/P3) and (iii) prophage-free chromosome (P1/P4) when the prophage is excised. Colony PCR analysis (Fig. 8) of the three wild-type strains shows that: (i) the P1/P2 as well as P3/P4 primer pairs yield an amplicon in the wild type strains verifying the correct predicted location of the prophage on the bacterial chromosome; (ii) P2/P3 amplicon, indicating the presence of circular prophage species in non-induced wild type strains; and (iii) P1/P4 amplicon, detecting the chromosome with excised prophage, which was found to be present in non-induced wild type strains too. The latter two observations confirm our hypothesis that the prophages are continuously excised and replicating even without induction.

In contrast, only P1/P4 primer pair yielded a PCR product for cured strains. P1/P4 sites are distant from each other by ~ 40 kb if the prophage is present on the chromosome, thus no product is amplified in the PCR reaction. If the prophage is excised from the chromosome a short fragment (396 and 196 base pairs for TIFN1 and TIFN2 respectively) is predicted and observed. The colony PCR results for TIFN4 and its cured derivative TI4c (not shown) were exactly the same as TIFN2/TI2c. This confirms the specificity of the primers as well as successful curing of the three strains.

### The prophages contribute to the host competitive advantage as revealed in competition experiment

**Fig. 9:**
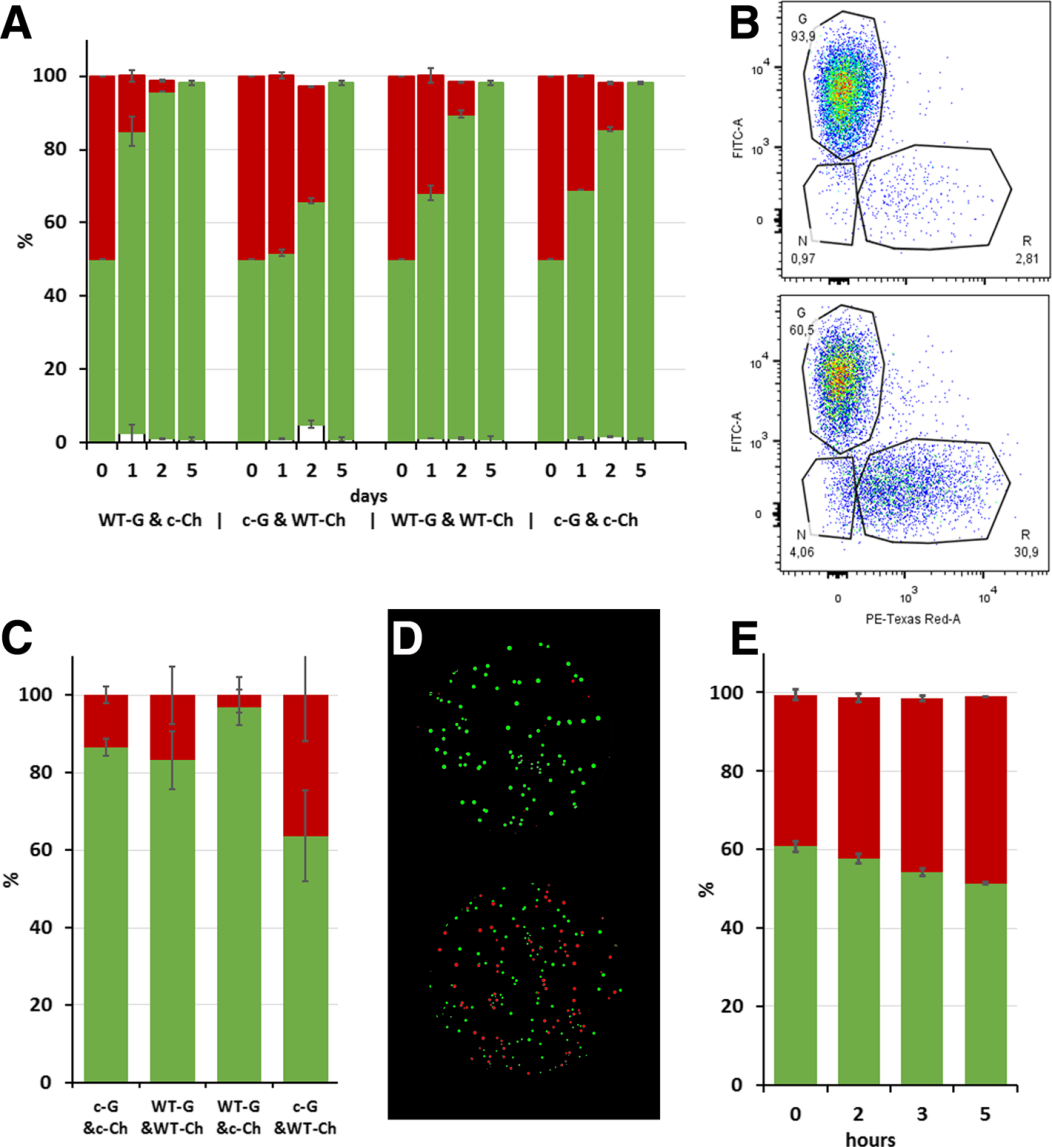
Competition between labelled wild-type and their prophage-cured derivative strains. **a** Ratio between labelled TIFN1 and TI1c cells propagated in mixed cultures in four different combinations and monitored by flow cytometry during 5 days. Green bars – GFP, red bars – mCherry, WT – wild type, c – cured derivative, G – GFP, Ch – mCherry. **b** Exemplary dot plots of experimental specimens shown in panel **a** recorded on propagation day 2 with encompassing gates for cell population expressing GFP-G, mCherry-R, negative cells-N and corresponding statistics; GFP-WT & mCherry-cured (upper panel) and GFP-cured & mCherry-WT (lower panel). On the y-axis is the GFP fluorescence intensity, on the x-axis – mCherry fluorescence intensity (log-scales). **c** Ratio between the GFP and mCherry expressing TIFN1 and TI1c cells after 2 days of propagation in mixed cultures as revealed by fluorescent colony count. **d** Exemplary images (overlay, pseudo colour) of the experimental specimens shown in panel **c**. GFP-WT & mCherry-cured (upper panel) and GFP-cured & mCherry-WT (lower panel). **e** Ratio between GFP-TI2c and mCherry-TIFN2 grown in mixed culture monitored by flow cytometry during 5 h

To further elucidate the impact of carrying prophage sequences on host fitness we designed competition experiments in which the wild type strains and the prophage-cured derivatives were labelled with either GFP or mCherry fluorescent proteins, encoded on plasmid and expressed from an artificial constitutive promoter. The GFP and mCherry expressing strains were mixed 1:1 in different combinations in duplicate and propagated in mixed cultures in LM17 medium. The cultures were diluted daily 1:100 and monitored during 5 days. To determine the change in the ratio between GFP- and mCherry-expressing cells in time 10,000 individual cells from each time point were analysed by flow cytometry (Fig. 9a). Remarkably, expression of mCherry in lactococcal cells had a detrimental effect compared to the expression of GFP. The control combinations of both wild types (WT-GFP & WT-mCherry) as well as both cured strains (cured-GFP & cured-mCherry) showed the same pattern, GFP expressing cells overgrew mCherry expressing cells. After 24 h of growth the ratio GFP/mCherry expressing cells in both control cultures were 70%/30%, by day 2 the ratio shifted to ~ 85%/15% and only GFP expressing cells remained in the cultures on day 5. In contrast, the experimental samples – the combination of WT-GFP & cured-mCherry and cured-GFP & WT-mCherry showed pattern of change different to each other as well as different to the control samples. mCherry-labelled cured cells were overgrown by the GFP-labelled WT much faster than the controls, whereas mCherry-labelled WT cells were relatively more competitive with the GFP-labelled cured strain. These data show that even under optimal growth conditions the prophage bearing wild type TIFN1 cells have a competitive advantage compared to their cured derivatives. Figure 9b shows the dot plots obtained for two exemplary experimental samples recorded after 2 days of propagation in mixed culture.

The same mixed cultures were diluted and plated after 2 days of propagation and the ratio between GFP and mCherry expressing cells were analysed by fluorescent colony count. The results corresponded to the results obtained by flow cytometry. On day 2 the GFP/mCherry expressing cells were ~ 85%/15% in both WT and both cured strains, 97%/3% in GFP-WT & mCherry-cured culture and 62%/38% in the combination of GFP-cured & mCherry-WT.

A growth experiment with a mixed culture was also performed with mCherry labelled strain TIFN2 and its GFP labelled derivative strain TI2c. The ratio of GFP and mCherry cells was monitored by flow cytometry during 5 h (Fig. 9e). A similar trend of wild type persistence in the culture compared to its prophage cured derivative was observed.

## Discussion

In the current study we investigated the presence, identity, behaviour, and impact on host competitive power of prophages in *L. lactis* strains isolated from a complex starter culture (Ur), used for industrial cheese production. All lactococcal strains analysed were found to be lysogenic. Remarkably, the prophages were found to be excised from the bacterial chromosome, replicated, packaged, and finally released from the cells in substantial amounts, both in the absence and presence of a prophage inducing drug (mitomycin C), although no tailed viral particles were detected in any of the supernatants from induced cultures. Earlier studies reported up to 10^4^ [35] and 10^6^ [36] plaque forming units per ml were present in supernatants of lactococcal cultures due to spontaneous induction. Our results implicate that between 10^8^ and 10^9^ (pro) phage particles per ml culture of the lysogenic strains are permanent residents of the microbial community of the starter culture, a number that is expected to increase further if the culture is subjected to environmental stress conditions such as elevated temperature or increased salinity. The latter conditions are encountered during cheese making. Some studies performed in factories where *L. lactis* is used as starter culture, reported up to 10^9^ phages per ml of whey e.g. [37] but this was regarded as a detrimental phage contamination. Interestingly, continuous or even induced release of prophages does not seem to result in massive cell lysis, not in the pure cultures of the isolates and also not in the complex starter [20].

The question now arises how the maintenance of lysogeny, and more particular the observed continuous release of phage crop, relates to the evolutionary success of the native strains in the undefined starter culture. Moreover, it should be noted that the abundantly released phage particles are tailless which may suggest they are incomplete or defective. Production of phage particles is an energetically expensive process as metabolic energy is required for the replication of the viral DNA and assembly of the virions. The process of phage DNA packaging is driven by a molecular motor that utilizes energy derived from ATP hydrolysis, as reviewed in [38]. For example, the force involved in the DNA compaction process for filling a phage capsid can be converted into an intra-capsid pressure as large as 60 atm at the expense of ATP hydrolysis [39]. It has been shown for Φ29 and T3 bacteriophages that 2 bp are packaged per ATP molecule cleaved by the motors [40, 41]. Despite this huge metabolic burden associated with the continuous release of phage particles, lysogens are obviously competitive in the community as initially evidenced by their high relative abundance in the culture and also confirmed in competition experiments between wild types and their prophage-cured derivatives. In mixed cultures the wild type lysogens outgrow the cured competitor, however, our experiments cannot discriminate between true increased fitness of the wild type strain from a detrimental effect of released phage particles on the cured competitor strain.

Although the phenomenon of spontaneous prophage induction (i.e. prophage induction under non-inducing conditions) in a microbial population has been described in the early 50s of the twentieth century [42] it regained attention in the recent years [43, 44]. Spontaneous induction is usually linked to the activity of an SOS response system, common in bacteria [45]. It is therefore not surprizing that for some strains we observed increased spontaneous induction in cultures challenged by stress conditions such as elevated temperature and osmotic upshift. In contrast, low nutrient concentrations can stabilize lysogeny [46] which also agrees with our observations that induction frequency in some cases declined. Remarkably, other authors have also observed enhanced induction of temperate lactococcal bacteriophage phiLC3 by environmental stimuli [47]. The authors found significant increase in induction frequency at elevated temperatures (34.5°C), which is in agreement with our observations. However, under the conditions of nutrients depletion (0.4-fold LM17) the authors observed an increase in phiLC3 induction as well, whereas elevated NaCl concentration (1.5%) had an opposite effect. This discrepancy indicates that the environmental stresses triggering prophage induction are phage or strain specific. It has also been observed earlier, for example, that prophages of the polylysogenic *Enterococcus faecalis* strain V583 responded differently to environmental challenges [48].

Spontaneous prophage activity has been studied also in models for biofilm formation, and pathogenesis of human diseases and it has been shown to lead to competitive advantages and benefits for the bacterial populations by enhancing biofilm formation, playing a vital role in bacterial virulence or leading to horizontal gene transfer [43, 49].

Our findings open new perspectives in understanding population biology and evolution of complex mixed microbial communities. Despite a huge metabolic burden imposed on cells due to continuous production and release of phage particles, the evolutionary success of these lysogens is evident from their high abundance in the culture. However, the most interesting question about the mechanisms by which the tailless (pro) phages provide their hosts with a competitive advantage remains to be answered. Furthermore, our findings suggest that the presence of prophages and their release plays an important role in the ecosystem.

## Conclusions

The results of this study indicate that chromosomal genetic elements are active participants in the stable complex microbial community, shaped by the natural evolution (and natural selection). We show that prophages are abundant in such a community, produced continuously in large amounts and, despite the huge metabolic burden imposed on the cells by phage particle production, provide a selective advantage to the host.

## Additional files

Additional file 1: **Figure S1.** Phage genome size estimation using conventional long run and field inversion (FIGE) agarose gel electrophoresis. A. proΦ1 (lane 4, non-induced, Rf = 0.167, 40.7 kbp), proΦ1 (lane 5, MitC induced, Rf = 0.167, 40.7 kbp) and proΦ2 (MitC induced, Rf = 0.173, 39.5 kbp) genomic DNA resolved using long run conventional electrophoresis. B. proΦ1 (Rf = 0.102, 40.7 kbp) and proΦ5 (Rf = 0.102, 40.7 kbp) genomic DNA resolved using FIGE. The λgenome (last lane) size, determined in the analysis is 48.3 kbp (Rf = 0.089), which is close to its actual size 48.5 kbp. C and D show calibration curves for A and B respectively. The size of the marker fragments (Thermo Scientific, in base pairs) are: λmix 19–48,502, 38,416, 33,498, 29,946, 24,508, 23,994, 19,397, 17,053, 15,004, 12,220, 10,086, 8614, 8271; High Range (HR) - 48,502, 24,508, 20,555, 17,000, 15,258, 13,825, 12,119, 10,171. (TIF 5860 kb)

Additional file 2: **Figure S2.** Phage DNA in supernatants of Mitomycin C induced 14 strains of TIFN1 and TIFN5 lineages visualized on agarose gel electrophoresis. High range ladder (HR, 48–10 kbp) and different λDNA concentrations are used as markers for molecular weight and phage release estimation. The lower molecular weight species on the bottom of the gel in **Figure S2** are presumably rRNA. Such bands, found in all phage preparations, correspond to ~ 1100 and ~ 900 base pair (DNA size), degraded by Benzonase® Nuclease but not by DNAse, and prone to degradation in time (not shown). (TIF 3020 kb)

Additional file 3: **Figure S3.** Spontaneous prophage induction in cultures of 7 TIFN strains subjected to stress conditions based on quantification of phage DNA by agarose gel electrophoresis. MitC driven induction and induction without any stress applied (NC) were also analysed in the series of experiments for comparison. (TIF 3302 kb)

Additional file 4: **Figure S4.** Comparison of prophage yield under stress growth and standard growth conditions. Filled bars indicate statistically significant difference in prophage induction. We allowed the confidence interval 90%, *p* < 0.1 (unpaired double-sided t-test versus negative control) to consider the difference significant because of significant impact of even small difference in induction conditions on amount of phages released as exemplified in Fig. 4. (TIF 1405 kb)

## Abbreviations

FIGE: Field-inversion gel electrophoresis
FSC: Forward-scattered light
GAC: Glycine adapted cells
GFP: Green fluorescent protein
LAB: Lactic acid bacteria
LM17: M17 medium, supplemented with 0.5% lactose
MitC: Mitomycin C
OD_600_: Culture turbidity, optical density at 600 nm
PEG: Polyethylene glycol
PHAST: Phage Search Tool
SSC: Side-scattered light
TEM: Transmission electron microscopy
WT: Wild type
