# Supplementary figures and images for: Spontaneously induced prophages are abundant in a naturally evolved bacterial starter culture and deliver competitive advantage to the host
Source: BMC Microbiol. 2018 Sep 24;18:120. doi: 10.1186/s12866-018-1229-1 (PMC6154921; doi:10.1186/s12866-018-1229-1)

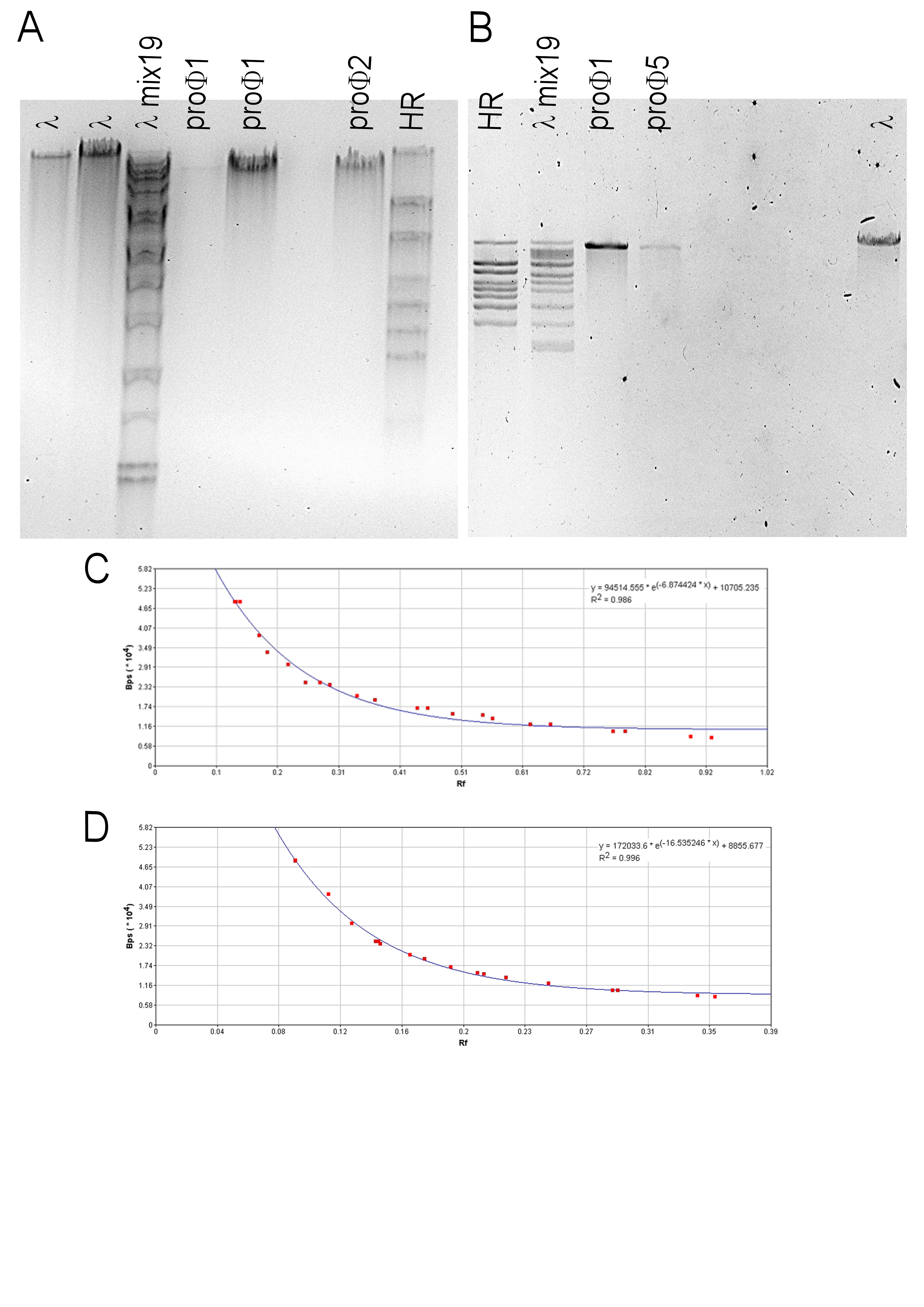

Supplement: Supplementary file 1 — Figure S1. Phage genome size estimation using conventional long run and field inversion (FIGE) agarose gel electrophoresis. A. proΦ1 (lane 4, non-induced, Rf = 0.167, 40.7 kbp), proΦ1 (lane 5, MitC induced, Rf = 0.167, 40.7 kbp) and proΦ2 (MitC induced, Rf = 0.173, 39.5 kbp) genomic DNA resolved using long run conventional electrophoresis. B. proΦ1 (Rf = 0.102, 40.7 kbp) and proΦ5 (Rf = 0.102, 40.7 kbp) genomic DNA resolved using FIGE. The λgenome (last lane) size, determined in the analysis is 48.3 kbp (Rf = 0.089), which is close to its actual size 48.5 kbp. C and D show calibration curves for A and B respectively. The size of the marker fragments (Thermo Scientific, in base pairs) are: λmix 19–48,502, 38,416, 33,498, 29,946, 24,508, 23,994, 19,397, 17,053, 15,004, 12,220, 10,086, 8614, 8271; High Range (HR) - 48,502, 24,508, 20,555, 17,000, 15,258, 13,825, 12,119, 10,171. (TIF 5860 kb) [file 12866_2018_1229_MOESM1_ESM.tif]

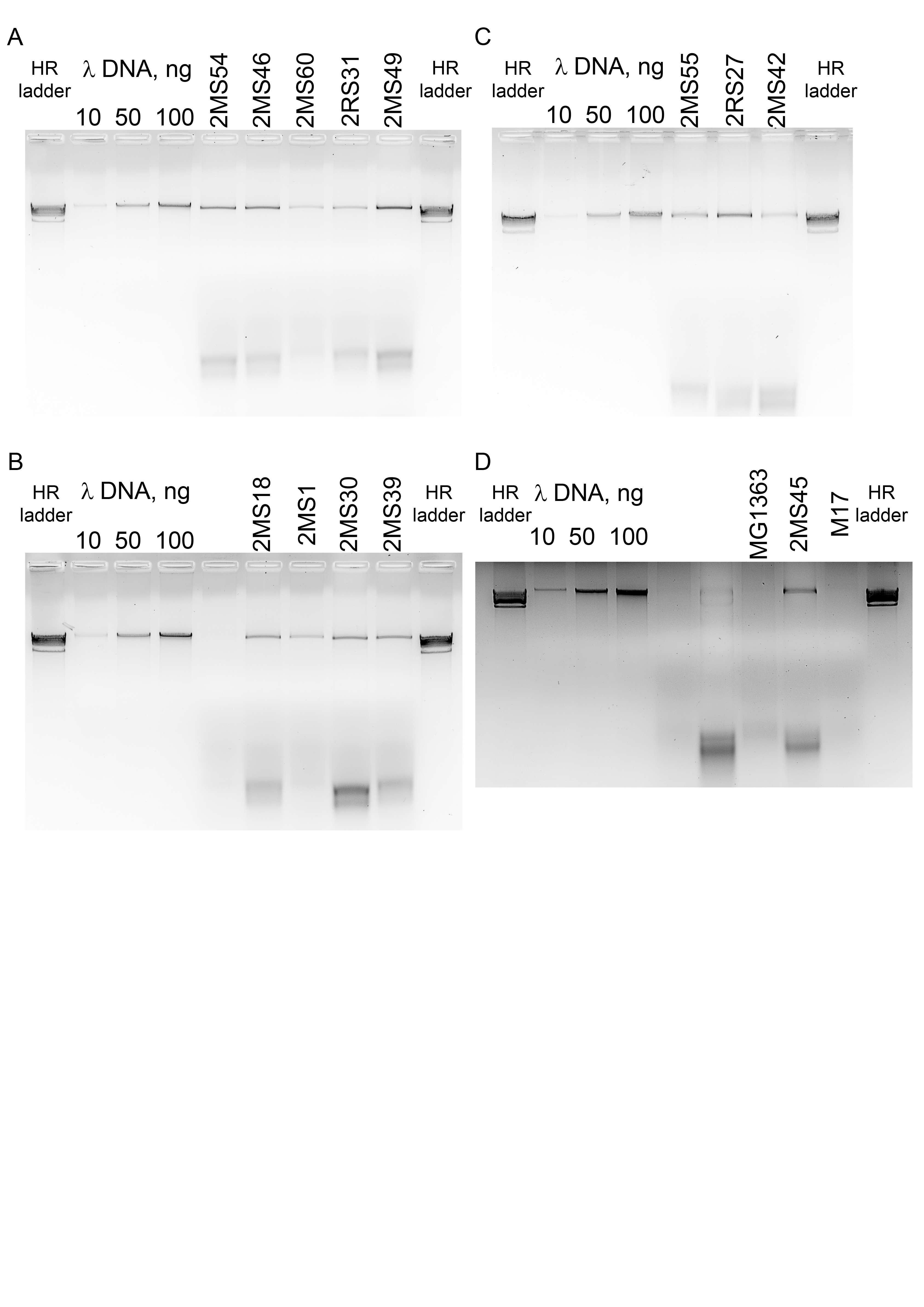

Supplement: Supplementary file 2 — Figure S2. Phage DNA in supernatants of Mitomycin C induced 14 strains of TIFN1 and TIFN5 lineages visualized on agarose gel electrophoresis. High range ladder (HR, 48–10 kbp) and different λDNA concentrations are used as markers for molecular weight and phage release estimation. The lower molecular weight species on the bottom of the gel in Figure S2 are presumably rRNA. Such bands, found in all phage preparations, correspond to ~ 1100 and ~ 900 base pair (DNA size), degraded by Benzonase® Nuclease but not by DNAse, and prone to degradation in time (not shown). (TIF 3020 kb) [file 12866_2018_1229_MOESM2_ESM.tif]

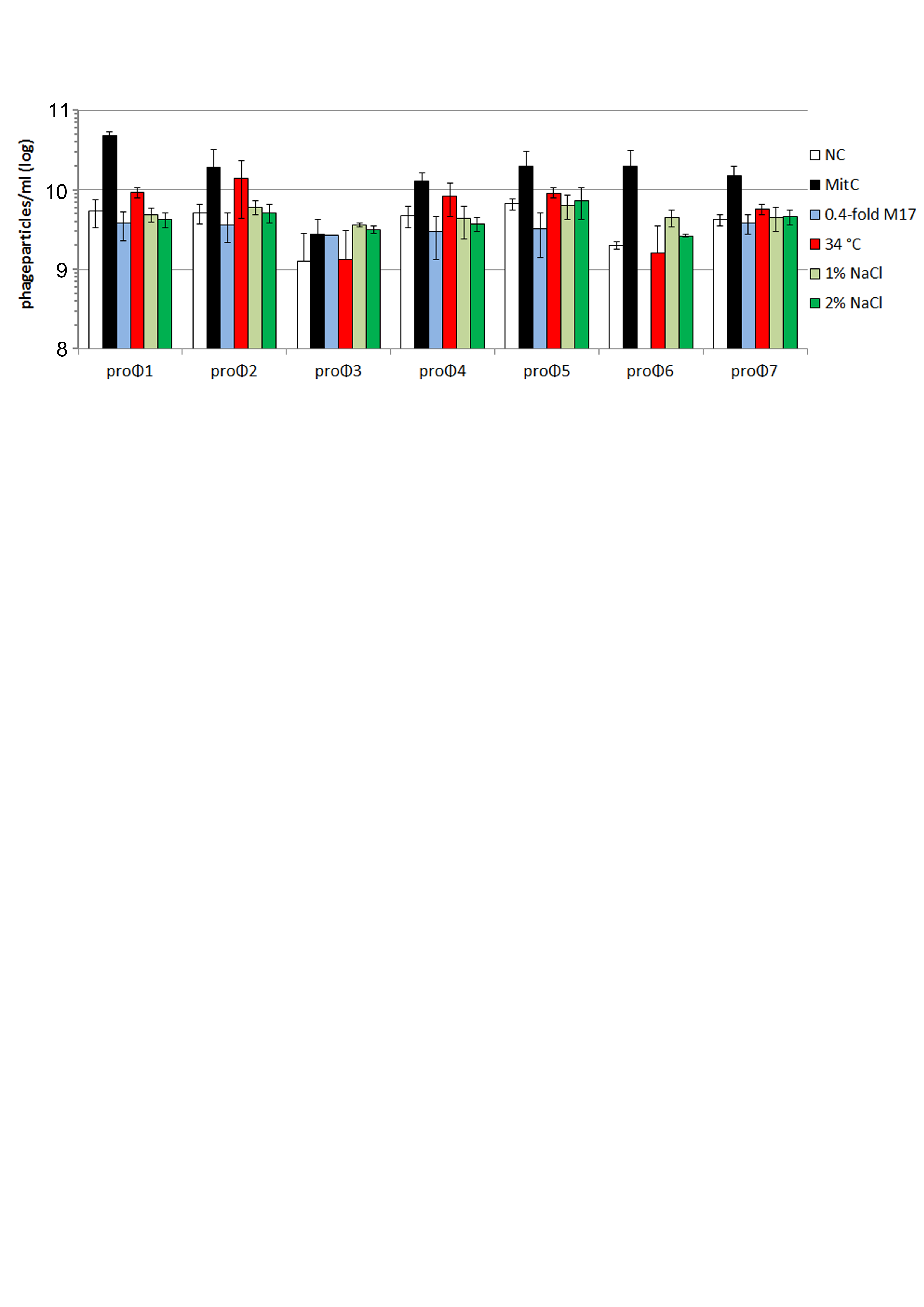

Supplement: Supplementary file 3 — Figure S3. Spontaneous prophage induction in cultures of 7 TIFN strains subjected to stress conditions based on quantification of phage DNA by agarose gel electrophoresis. MitC driven induction and induction without any stress applied (NC) were also analysed in the series of experiments for comparison. (TIF 3302 kb) [file 12866_2018_1229_MOESM3_ESM.tif]

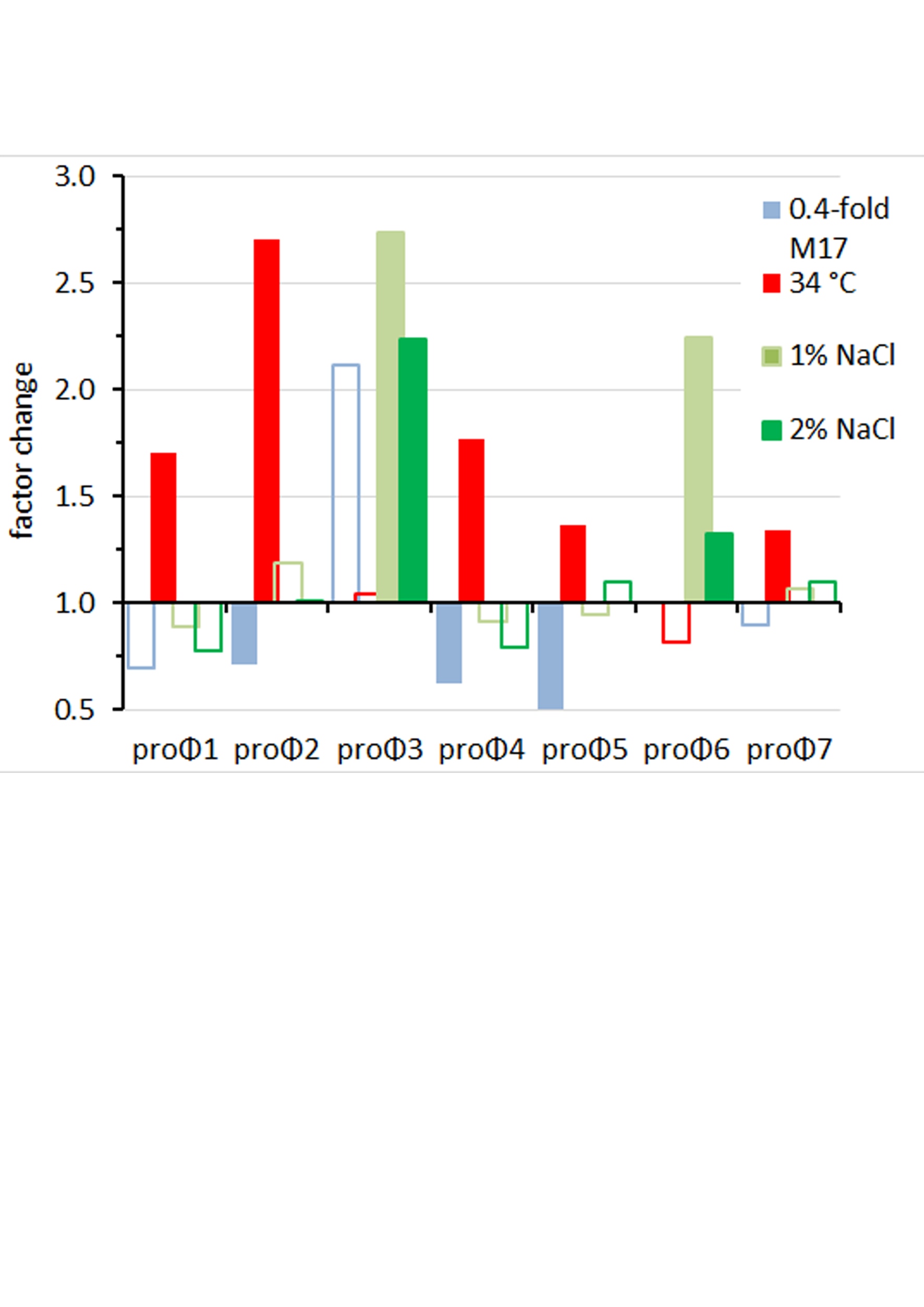

Supplement: Supplementary file 4 — Figure S4. Comparison of prophage yield under stress growth and standard growth conditions. Filled bars indicate statistically significant difference in prophage induction. We allowed the confidence interval 90%, p < 0.1 (unpaired double-sided t-test versus negative control) to consider the difference significant because of significant impact of even small difference in induction conditions on amount of phages released as exemplified in Fig. 4. (TIF 1405 kb) [file 12866_2018_1229_MOESM4_ESM.tif]
